# EMS operation in a female restricted university, Saudi prospective

**DOI:** 10.1186/s12245-021-00339-8

**Published:** 2021-02-24

**Authors:** Wajdan Alassaf, Sameer Al Hamid, Osama Kentab, Abdullah Al Otaibi, Bandar Al Mufareh

**Affiliations:** 1 Emeregncy Department, College of Medicine, Princess Nourah Bint Abdulrahman University, Riyadh, Kingdom of Saudi Arabia; 2 Emergency Department, King Abdullah Bin Abdulaziz University Hospital, Princess Nourah Bint Abdulrahman University, Riyadh, Kingdom of Saudi Arabia; 3 Royal Commission Hospital, Jubail, Kingdom of Saudi Arabia

**Keywords:** Emergency medical services, Collegiate emergency medical services, Restricted female university campus

## Abstract

This paper was written to explain the process and steps and to describe the experience with building a women-only operated collegiate emergency medical service (EMS) system in the largest women-only university in the world. To the best of the authors’ knowledge, the EMS system described in this report is the first collegiate EMS system in the Gulf region. The concept of the collegiate EMS system at the university, the factors that mandated the creation of this system, the process steps, the challenges faced, and, finally, the reported outcome have been evaluated. The women-only campus conferred unique challenges and additional pressure during the planning and implementation stages of this project; our system had helped in decreasing response time to medical emergency, provided back up support during mass gathering events in the university, and helped in decreasing the load on other national EMS services.

## Introduction

Emergency medical service (EMS) is defined as “a comprehensive system that provides the arrangements of personnel, equipment, and facilities for the effective, coordinated, and timely delivery of health and safety services to victims of sudden illness or injury” [1], and the EMS is strongly recommended and supported by the World Health Organization (WHO), which gives it a role in both healthcare systems and in supporting the individual’s right to healthcare access [2]. Moreover, EMS is recognized as a subspeciality by both the American College of Emergency Physicians and the Australian Medical Council [3].

The concept of collegiate-based emergency medicine services (CBEMS) is a new system whose origins can be traced to 1948, and individual groups have been previously reported to provide CBEMS [4, 5]. The response time in some colleges was less than 3 min because of the CBEMS [6].

There are 11 and 134 CBEMS in Canada and in the USA, respectively [7]. However, Saudi Arabian universities did not implement the concept of CBEMS until 2014 when we started our own system. The Princess Nourah Bint Abdulrahman University (PNU) campus is located in Riyadh, Saudi Arabia, with over 8 million M^2^, 600 buildings, 766 classrooms, 35 lecture halls, and 3 high-tech auditoriums. More than 40,000 students of approximately 60 nationalities study at the university, and together with the faculty and administrative staff, the total population in the university campus exceeds 60,000. The university campus provides residential facilities for both faculty and students. The King Abdullah Bin AbdulAziz University hospital (KAAUH) is the academic hospital serving the university, allocated within the campus.

The college campus (hereafter referred to as “the campus”) is a restricted, women-only campus. Prior to the implementation of the novel EMS system, the university used to rely on the Saudi Red Crescent Society (SRCS) for emergency cases, and, given that all of the EMS personnel in the SRCS were males, this affected their access to the campus and prolonged the response time. Additionally, campus entry was restricted and permitted only for faculty, employees, and students; all other individuals who intended to enter the campus had to apply for a permit to seek access. The abovementioned restrictions applied to the students’ housing in the university campus. Emergencies were reported by the security personnel to the SRCS, who would dispatch an ambulance to transfer the patients; however, this particular response was associated with several risks (Table 1).

**Table 1:** Risks associated with the initial response system

Risks	Details
Initial response was provided by untrained personnel	Response was carried on by security personnel and volunteers who are not BLS trained, without any medical background
Difficult access	Only certain gates had service elevators that could accommodate stretchers
Permit is needed for the access to be opened during working hours	Entry to the colleges is restricted; only students, faculty, and administrative staff are able to enter the premises Initially, even hospital personnel had to obtain official access permits, for which the process may take up to 2 weeks
Lack of onsite medical response	The university medical center served patients on site only, with no response to any cases outside the clinics
Lack of medical response equipment	The campus lacks AEDs; some buildings had first-aid bags which comprised bandages and band-aids

AED, automated external defibrillator

A university medical center (UMC), managed by 4–7 general physicians from 8 am to 4 pm during weekdays, within the campus provided onsite treatment (serving patients with appointments and walk-ins), but they did not attend to emergencies outside the UMC. Accessing the UMC was difficult because of the long distances between colleges that mandated prolonged walking or use of the metro station; moreover, the UMC physicians had not been trained in family medicine or emergency medicine.

There is a low prevalence of knowledge of cardiopulmonary resuscitation (CPR) among Saudi citizens; a recent paper that reported the findings of a study on CPR knowledge among university students (PNU University) showed that 87.9% of the study sample had poor knowledge of CPR [8]. Other studies conducted among students studying at Saudi Arabian universities showed that basic life support (BLS) courses were incorporated in only 40.35% of the college syllabus. Nonetheless, good BLS knowledge was generally observed in both medical (61.2%) and nonmedical (53.2%) student participants, although this information was unavailable at the time that the plan was developed to design and implement the collegiate EMS system in the PNU [9].

Based on the abovementioned factors, the necessity of creating an EMS system in the campus to provide more appropriate care for the extended population was explained by the hospital administration to the university administration. This was particularly important in view of the fact that dependence on the volunteers, as is the case in the US colleges, was not feasible. Therefore, it was important to prepare an appropriate setting to create an emergency response base before encouraging the training of the rest of the staff or student volunteers.

In this paper, we describe the process of initiation of planning, testing, implementation, and troubleshooting before the collegiate EMS became fully operational, as well as the impact of this system on the quality of health care provided to the patients.

### Stakeholder’s meeting

Multiple needs assessments were conducted through discussions with multiple stakeholders, including the deans of different colleges, vice-rector of student affairs, security directors, and other personnel in addition to that with the students. We decided to direct efforts in three domains:

1. Urgent domain, which involved the recruiting of female paramedics to establish the human resource for our initial EMS stations and to supply the equipment they needed to perform the tasks required from them.
2. Semi-urgent domain, which included the training of security personnel in first-aid and CPR to establish consistency of care provision and to support the female paramedics.
3. Future domain, which comprised educating student teams on first-response techniques and supplying the university with automated external defibrillators (AED) in different locations on the campus to support urgent response.

All extracurricular university activities in addition to annual events that involved mass gatherings, such as the registration of new students, were dependent on the hospital’s medical support, wherein the hospital or the UMC used to send their physicians and nurses to be available during such gatherings. Besides being cost effective, the EMS was meant to alleviate the increased burden on the hospital because of the deployment of their staff to provide support within the campus via the EMS stations. Prior to securing the locations of the dedicated EMS stations, the female paramedic teams were directed to serve the university and all its related activities by including them in the UMC as well as in the hospital, where their role was to respond to cases outside the UMC and, afterward, to either transport them to the UMC or the hospital based on their assessment of the clinical care requirements and the directions of the emergency medicine consultant.

### Initiation

After consideration of the needs as well as the goal to establish the commitment to health and safety in the university and the continuous, multiple facility expansions in the university that required medical attention, such as the gym, football stadium, running stadium, new administrative offices, and the multiple previous complaints of delays in the provision of emergency care at meetings with the stakeholders, the project received the green light to commence the planning stage. The planning started with a tour of the PNU to determine the places where the EMS stations needed to be established.

The objectives of this project were:

- To deliver outstanding and confidential prehospital patient care to the university community
- To plan and execute special operations in collaboration with other departments to ensure the safety of the campus, its residents, and visitors
- To educate and assist the university community in emergency preparedness, including CPR, first aid, personal safety, and disaster readiness
- To create an environment of care that encourages diversity and advancement among all hospital EMS personnel and supports the fulfillment of individual goals as well as respect for core values and accountability

**Fig. 1:**
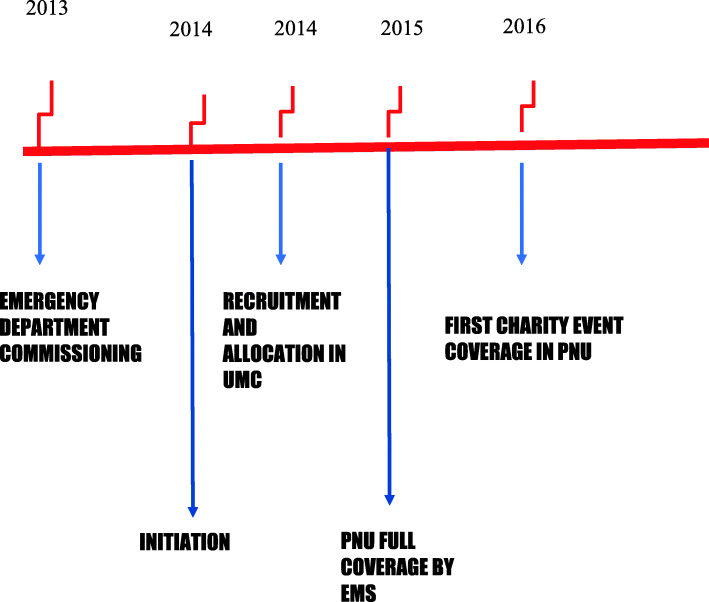
Time line of CEMS development in PNU

In the initial proposal (phase I), only the setting up of the emergency call response was planned to ensure inter- and intra-facility transportation that would require reliance on external agencies until phase II of the project. Preparedness for disaster response and mass gatherings were included in phase I as were public health education campaigns. This part of the project was completed in a phased manner in multiple timeframes (Fig. 1).

The implementation process was long and difficult, required more than 1 year to complete, and was hampered by difficulties with major budget issues and communication.

### Recruitment

The major universities that provided graduate programs in emergency medical systems for women were personally contacted, their list of graduates were obtained, and suitable candidates were contacted via telephone and invited for interviews if they expressed an interest to join the EMS. In total, five female paramedics and seven male personnel (EMTs and paramedics) were recruited and commissioned to work in the Emergency Medicine department. Initially, the female paramedics worked for a few months in the hospital to familiarize themselves with the EMS system and the hospital facilities. Then, they were assigned to the UMC, where they were to respond to calls as advanced life support (ALS) units with stretchers, defibrillators, and BLS medications and equipment in the response bags (Table 2), while the male team was assigned to work in the EMS department in the hospital itself, operating the dispatch center, and driving ambulances to transfer cases either from the university to the hospital, or from the hospital to other receiving centers (women driving pan was lifted in 2018).

**Table 2:** Response bag components

Bag compartment	Components
Outer front compartment (airway)	OPA, NPA, suction catheter, Yankauer suction
Outer left compartment (breathing)	Oxygen nasal cannula, adult & pediatric; face mask, adult and pediatric; nebulizer kit, non-rebreather masks, adult & pediatric; oxygen tubing
Outer right compartment (PPE)	Disposable gloves, disposable mask, goggles, isolation gown, sterile gloves, hand sanitizer
Inner top-left pouch (misc.)	Band-aid, eye pad, triangular bandage, thermal blanket, trauma shears
Inner top-right pouch (IV supply)	IV cannula, butterfly cannula, syringes of all sizes, needles of all sizes, IV set, IV dressing, gauze 2 × 2, hypoallergenic plaster, Betadine Swabsticks
Inner main compartment 01	Bag–valve–mask device
Inner main compartment 02 (dressing supply)	Nursing pack dressing, gauze 4 × 4, irrigating solution, Betadine Spray, underpads, SAM Splint, armsling, Chest Seal
Vital signs monitoring	Digital thermometer, BP apparatus, adult, pulse oximeter
Inner main compartment (medications)	0.9% normal saline (500 mL), Ringer’s lactate (500 mL), D5 water (500 mL), activated charcoal suspension, albuterol, MDI, Atrovent nebule, chewable aspirin; epi-pen, adult; epi-pen, pediatric; ibuprofen tablet, paracetamol tablet, nitroglycerin tablet, Flamazin gel, Voltaren gel

*PPE* personal protective equipment, *MDI* metered drug inhaler, *IV* intravenous, *OPA* oropharyngeal airway, *NPA* nasopharyngeal airway

## Education

The science graduate program in emergency medical systems for women is a recently introduced program in Saudi Arabia. As a consequence, the newly recruited female paramedics were fresh graduates without any prior clinical experience. We started an educational program that included lectures and practical training for the entire paramedical team and was conducted by senior registered nurses with more than 10 years’ experience in a trauma center emergency department as well as by board-certified emergency physicians with an EMS fellowship. The training was initiated from receiving calls to logging in the details in the system and to providing on-site response and management.

In comparison with other CBEMS, the EMS service is provided entirely by ALS-certified paramedics whereas ALS was provided in only 10% of the EMS service in other CBEMS because the majority of the personnel are student volunteers [4]. As part of the planning for phase 1, many training and educational sessions, such as heart-saving courses, were to be conducted for the security and university staff.

### Equipment

The need for equipment and supplies was calculated, including the requirement for back-up capital equipment, which was to be used for replacement in case of maintenance-related issues and to facilitate the EMS response in mass gatherings (Table 3).

**Table 3:** Total requested station’s equipment

Equipment	Quantity
Lifepak15 (monitor/defibrillator)	4
Golf-cart/mini-ambulance	3
Segway PT mobile rescue	4
Thermometer	4
Glucometer	4
Back boards	8
Cervical collars	16
Walkie talkie	9
Oxygen cylinders	6
Stretchers	8
BLS bag	4
ALS bag	4
Portable suction devices	4

*ALS* advanced life support, *BLS* basic life support

#### Supplies

Supplies were to be provided by the hospital. For this, the EMS team in the PNU had to follow the standard process of requesting, stocking, and replacing supplies similar to that by other hospital departments. However, transporting supplies to the response stations was challenging as cars were not allowed in the campus. Before the golf carts were made available, the team used to transport supplies in stretchers or in folding shopping carts.

#### Dispatch and communication

A dedicated helpline for reporting campus medical emergencies was set up, and the calls were received by the hospital EMS dispatcher, who initially would obtain a brief history and the exact location before either directing the caller to the UMC or dispatching the closest EMS unit to the location of the patient.

### Stations, equipment, and operation

#### Allocation of stations

**Fig. 2:**
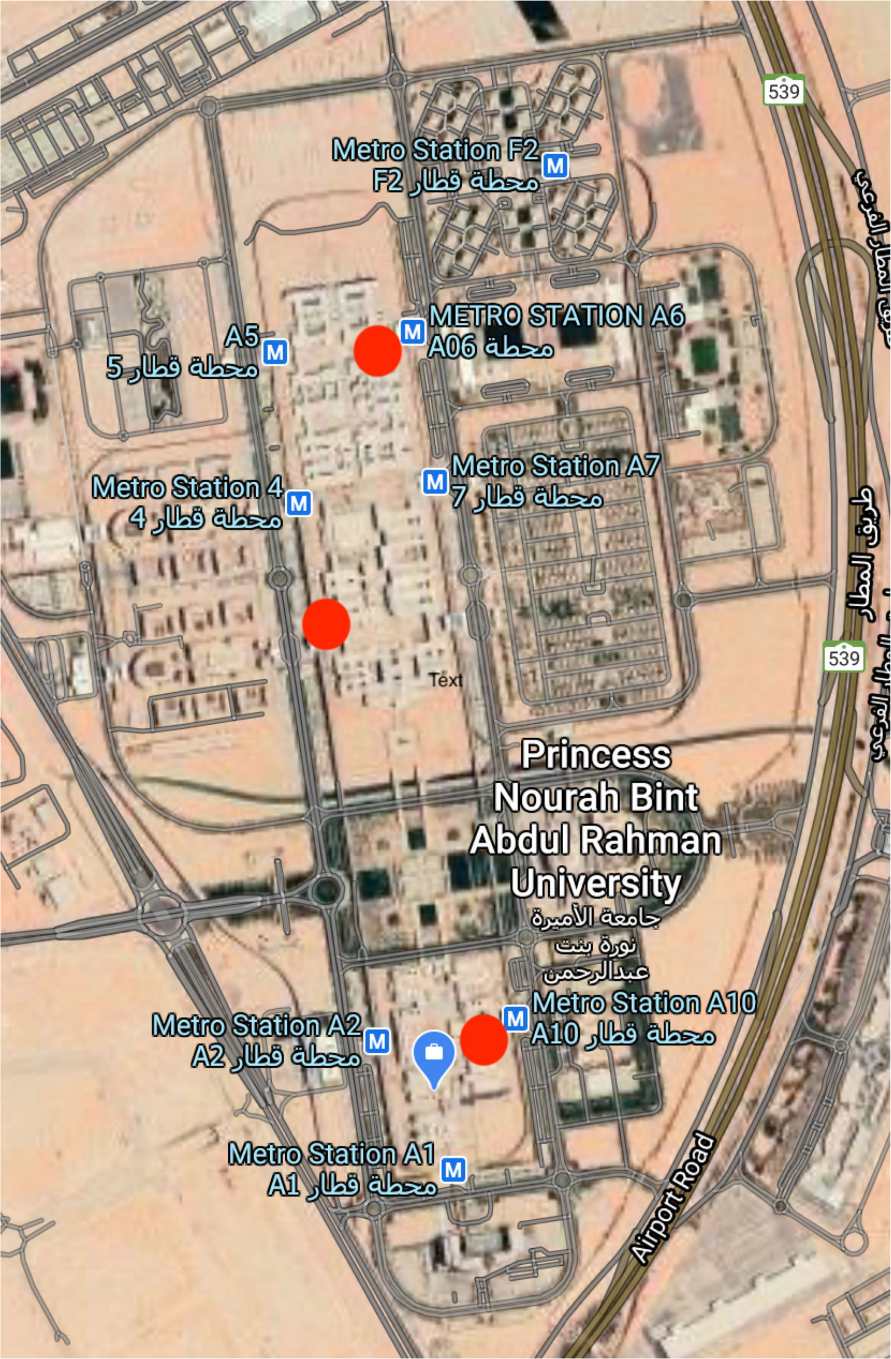
Campus based EMS stations (red circles)

After multiple site visits, an initial proposal for five EMS stations was submitted; however, approval was received for only three stations from the hospital’s Chief Executive Director and the university rector due to financial and space-related constraints (Fig. 2).

These station locations were situated to facilitate access to all colleges and to reduce the response time.

**Fig. 3:**
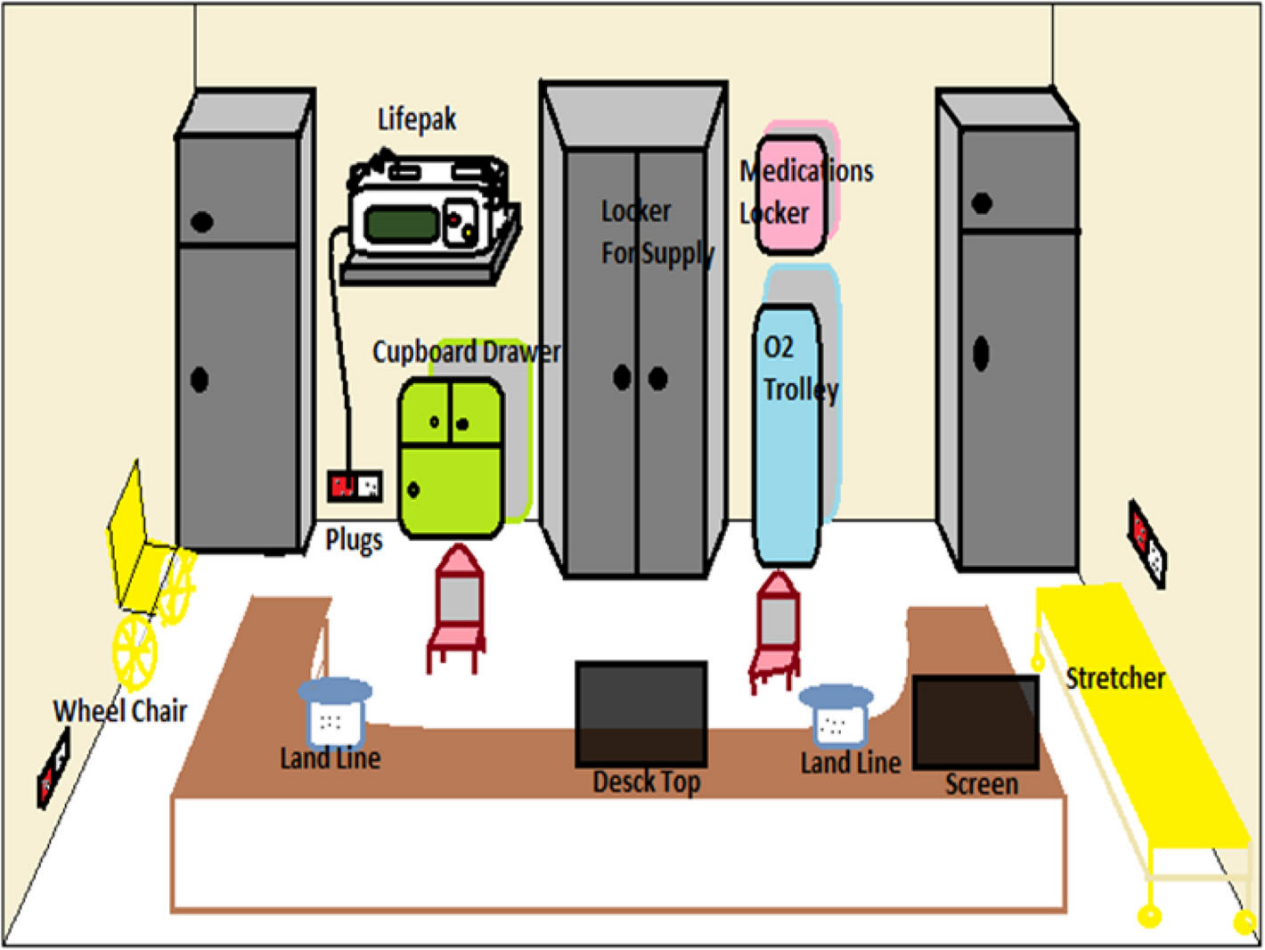
Stations layout

All EMS stations were designed identically (Fig. 3). The decision was made to move the new EMS team to dedicated stations instead of the UMC after the team had obtained a reasonable amount of experience in the university layout, shortcuts, and location of the colleges. After a careful and thorough discussion, the female paramedic teams were allocated to three different locations inside the university campus—at the A3, A6, and A10 stations, based on the population density, ease of access to external assistance if needed, and an aim to achieve the shortest possible response time (Fig. 1).

The stations were equipped with computers, telephones, and communication radios. Each team had an ALS response bag that contained the basic and essential medications and equipment (Table 2). The main dispatch center was in the hospital emergency department, and the emergency calls were received through a dedicated and unified emergency helpline number. The teams were usually dispatched after they received a call from the hospital dispatch center. A total of three Club Cars with stretchers were used to transport the patient to the medical center within the university or to meet the ambulance crew at the nearest gate, from where the patient could be transported to the hospital as necessary. The team underwent multiple practical training sessions on driving, parking, and charging the golf carts (this system was initiated before the new women’s empowerment movement in 2018 by the government to allow women to drive).

On arrival at the scene of the emergency, the team would assess the patient’s condition, initiate the first-responder management, and communicate with the medical care director—the on-call emergency medicine consultant—to decide whether to transfer the patient to the hospital or to the UMC, to be treated there by the respective medical teams. Most of the minor cases would be managed and discharged at the scene by the female paramedic team.

During summer vacations when the university has no students, the teams are reassigned to the main emergency department of the hospital to work with the rest of the EMS staff in the hospital that helped to transport patients.

#### Medical control

The team operated on the basis of both offline and online medical directives as well as educational sessions and were provided instruction manuals that included a brief description of common emergency presentation and lines of management. A paper-based prehospital assessment form (Appendix) and refusal of treatment form were provided to the team in order to keep track of the case documentation. The EMS Medical Director (who is also the EMS section head) produced and regularly updated policies, standard operating procedures, and clinical protocols. In addition, a regular audit of patient report forms was conducted as part of a culture of continual quality improvement.

#### Costs

Each station’s equipment and supplies costed a total of 60,000 USD with a total of 25.600 USD as monthly salaries for the entire EMS team.

#### Challenges

Establishing a new EMS system is associated with many challenges, such as maintaining a professional workforce and infrastructure as they seek to meet the needs of their community. In this project, building an EMS system that was staffed by only female paramedics posed additional unique challenges. Funding was a major concern as there was no dedicated budget for such a large project. EMS providers include both male and female paramedics; however, the Saudi EMS was fully staffed by males until a few years ago. Only recently, a few universities started to offer structured EMS programs for female students. Thus, there were no experienced female Saudi Arabian paramedics who could be recruited directly to the EMS team. Furthermore, in their internship year, the female EMS students worked as nurses rather than paramedics, except at one center in the Kingdom of Saudi Arabia, and this factor limited their experience and initial efficacy in providing field-based management.

Recruiting and retaining first responders was another challenge and an essential element to maintaining the daily operations of the EMS system. The recently unified salary scale made it impossible to recruit experienced local EMS providers from other institutions because they were on the old pay scale, with all of the associated benefits.

Communications capabilities, supplies, and assets were other obstacles, as the demand increases although the supply remains the same for a long period. The limited number of golf cars and ambulances was an issue, and the need is higher than what could be met with the available resources. Other challenges included, but are not limited to:

- Changes to the stakeholders’ requests according to need
- Hiring of female EMTs
- The need for a female EMS coordinator and liaison between the university’s female population and the university hospital in case of emergency responses and transportation
- With Vision 2030, the operation is expected to become easier in terms of hiring expert personnel in EMS responses, wherein male staff can directly be involved in the women-only EMS system
- Vacation distribution as shortage had limited the number of employees eligible for vacation in each month
- Physical capabilities, given the small build of a majority of the female paramedics
- Loss of interest in EMS by paramedics who started looking for office jobs because of the lack of lucrative financial compensation
- Uncoordinated needs from the university to cover events at short notice
- Difficulty in registering patients from EMS teams in the system except at the end of the day
- Implementing education standards
- The lack of an electronic dispatch system
- Lack of interest in research and quality projects secondary to work burnout
- Variations in the staff training and education programs
- Human resources and manpower, as no new lines for recruiting more personnel were created
- Lack of a proper communication system or radio between the dispatch and allocated units
- Lack of a budget allocation for the maintenance of ambulances and golf carts
- Staff turnover and resignations

#### Operations

**Fig. 4:**
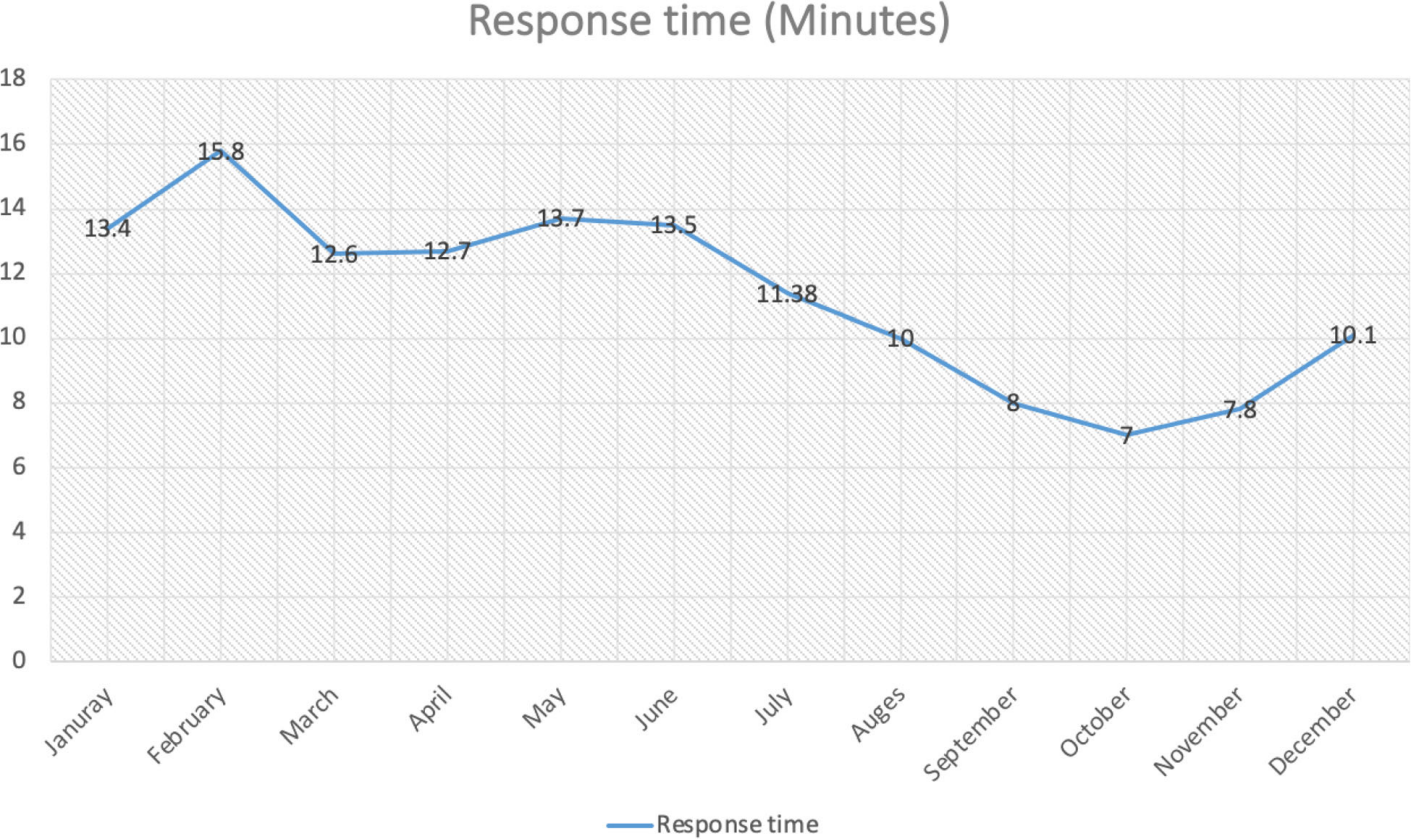
Response time analysis for 2019

**Fig. 5:**
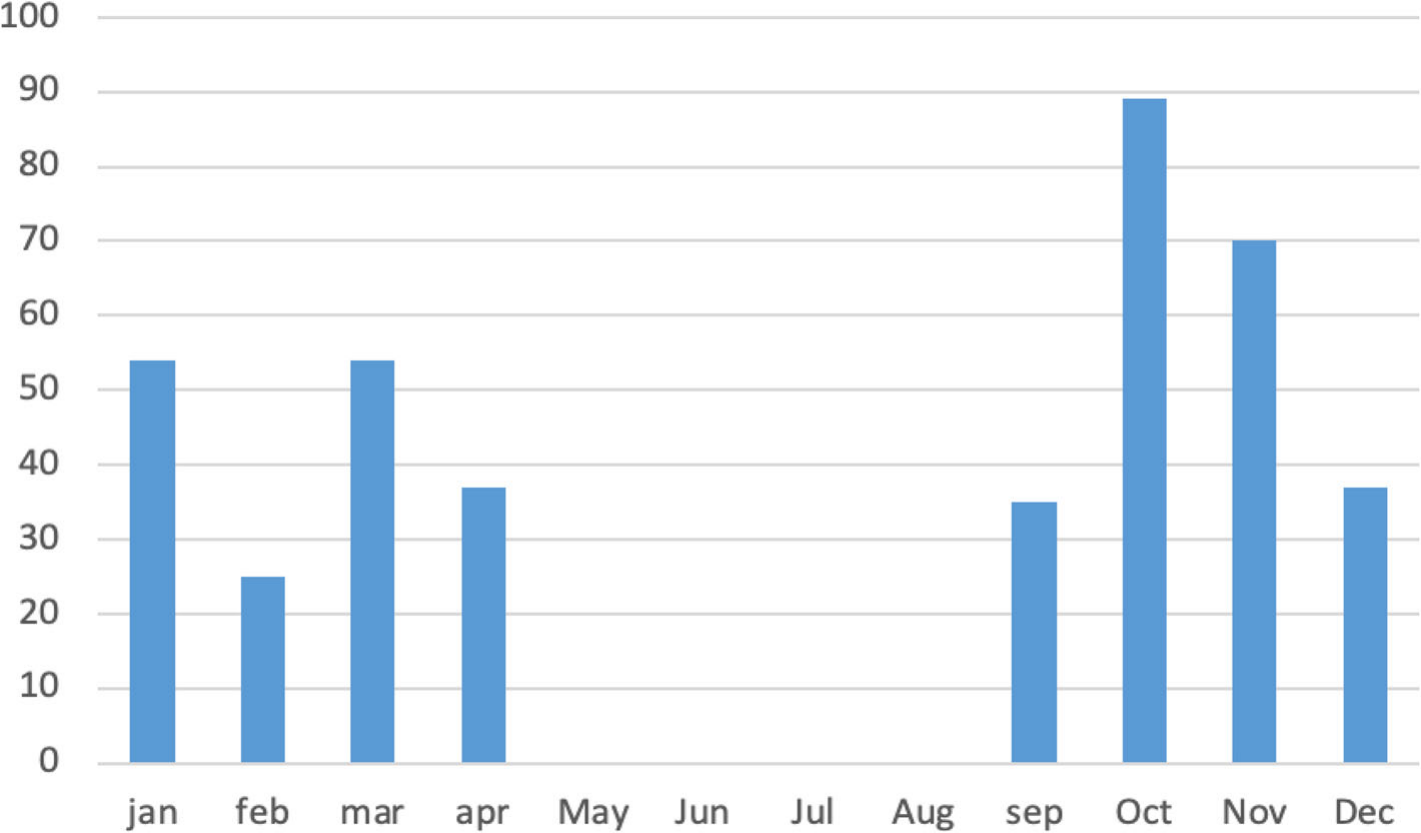
Cases transferred from PNU to KAAUH 2019

After the new EMS system became operational, the response time reached an international standard of 8 min, with the call volume and number of transferred cases amounting to 364 in 2019 (Figs. 4 and 5) in comparison to 17-min response time in 2018. Data for response times and quality of cases prior to the CBEMS initiation was difficult to collect as there were not a unified dispatch center and no pre-set quality indicators. At a specific timepoint, the team had to cover the student’s gymnasium and provide care for both trainers and trainees. After almost a year and with further data analysis (number of cases, acuity, and staffing-related concerns) the EMS team’s coverage of the gym center ended, and responses were provided only on call by the EMS system.

Quality is an integral part of the operations and, therefore, the operating team was divided into several teams, with each team holding a specific task and maintaining the quality of the work. The two major teams were the Medical Development Team and the Operation and Maintenance Team.

The Medical Development Team comprised:

1. Education coordinators
2. Research team
3. Clinical practice guideline preparation and quality team

The operation and maintenance team were constituted by the:

1. Ambulance maintenance supervisor and his team
2. Logistic and supply follow-up team
3. Team for dispatch quality

The teams usually comprise two or more members and are needed to perform the task and submit a weekly report to the section head about the tasks accomplished, pending tasks, and obstacles faced by the team. Therefore, the role of the manager is to facilitate the team’s mission and monitor its development.

Within several weeks, the team had set up an annual educational schedule and had begun to present and share the schedule with others such as airport paramedics/EMTs as part of a mutual agreement. Therefore, the KAAUH educational program became a source for educational activities that were directed toward EMS members. Moreover, workshops and first-aid courses were conducted to target community wellbeing. Thus, many clinical practice guidelines and policies have been written and reviewed by the quality team.

The dispatch development team set up the EMS helpline and provided the team with mobile phones that were connected to the helpline and preceded by communication in a bravo system of walkie talkies that were linked via a Wi-Fi extension through the PNU problem. They trained the call recipient on communication skills, starting with the welcome message and ending with patient advice and guidance.

During the administrative and clinical work burden, the wellness of employees remains an important factor that is measured annually. In the evaluated personnel, the level of burnout did not exceed 6%. In addition, the job stability rate proved reassuring.

## Data Availability

Not applicable.
